# Myocardial infarction: a critical role of macrophages in cardiac remodeling

**DOI:** 10.3389/fphys.2015.00107

**Published:** 2015-04-07

**Authors:** Tobias Weinberger, Christian Schulz

**Affiliations:** ^1^Medizinische Klinik und Poliklinik I, Klinikum der Universität, Ludwig Maximilians-UniversitätMunich, Germany; ^2^Munich Heart Alliance, DZHK (German Centre for Cardiovascular Research)Munich, Germany

**Keywords:** heart, development, myocardial infarction, macrophages, yolk sac, hematopoiesis, inflammation

## Abstract

Ischemic heart disease is a common condition and a leading cause of mortality and morbidity. Macrophages, besides their role in host defense and tissue homeostasis, are critical players in the pathophysiological processes induced by myocardial infarction. In this article we will summarize the current understanding of the role of monocytes and macrophages in myocardial damage and cardiac remodeling in relation to their origin and developmental paths. Furthermore, we describe their potential implications in therapeutic strategies to modulate myocardial healing and regeneration.

## Introduction

Cardiac diseases, especially acute myocardial infarction and congestive heart failure, are among the most frequent causes of death in the western world and are on the uprise in developing countries. Even though the survival after myocardial infarction has significantly improved by early revascularization therapy and drug treatment, a significant number of patients develop heart failure. A broad spectrum of local and systemic mechanisms are initiated in myocardial infarction and contribute to cardiac remodeling. If uncontrolled, they may lead to negative changes in the geometry, structure and function of the ventricle and thus may have deleterious effects on cardiac function in the long term. Therefore, great interest lies in the discovery of new therapeutic strategies to treat the conditions, which precede heart failure.

In myocardial infarction, occlusion of a coronary vessel (i.e., after coronary artery plaque rupture) results in acutely diminished oxygen supply to the myocardium, which leads to myocyte necrosis and acute inflammation. As the human heart has only limited regeneration capacity (Bergmann et al., 2009), infarct healing is–in addition to restoration of oxygen supply–very much dependent on the inflammatory response. Macrophages are an integral part of the innate immune response. They are equipped with an array of pathogen recognition receptors which can activate phagocytosis of pathogens and the secretion of cytokines and chemokines. They present antigens on their cell surface by constitutively expressed major histocompatibility complex II (MHC II) and thereby interact closely with the adaptive immune system.

In vertebrates, macrophages are found in all organs, where they adopt distinct phenotypes and functions that are shaped by the environment of the organ of residence (Gosselin et al., 2014; Lavin et al., 2014). Macrophages are also found in larger numbers in the mammalian heart (Jung et al., 2000; Epelman et al., 2014a; Heidt et al., 2014; Molawi et al., 2014).

Macrophages have long been thought to be of importance in cardiac remodeling after myocardial infarction in humans (Mallory et al., 1939). In autopsy reports macrophage numbers associated closely with different stages of myocardial infarction (Chang et al., 2013). Likewise, macrophages play a critical role in experimental infarction models including rodents (Yang et al., 2002) and swine (Vilahur et al., 2011). In this review we will summarize the functions of monocytes and macrophages in myocardial infarction and outline potential therapeutic strategies to improve infarct healing and outcome by manipulating distinct subsets of mononuclear phagocytes.

## Recruitment of monocytes into the ischemic heart

The immune response to myocardial ischemia can be divided into distinct but overlapping inflammatory phases, in a simplified view an early (3–4 days) and a late (7–10 days) phase. The kinetics are dependent on the pathological conditions and respective disease models (e.g., species analyzed, chronic ligation vs. ischemia and reperfusion model). Within 30 min after induction of infarction through ligation of the left anterior descending artery (LAD), monocytes are recruited to the site of injury as shown by *in vivo* microscopy (Jung et al., 2013; Nahrendorf and Swirski, 2013). However, the applied reporter mice did not permit distinguishing between the monocyte subsets involved. In mice, blood monocytes are divided into two principal subsets: Ly6C^hi^ CCR2^+^ CX3CR1^lo^ “inflammatory” monocytes (CD14^+^ in humans) and a less frequent subset of Ly6C^lo^ CCR2^−^ CX3CR1^hi^ monocytes (CD14^dim^ CD16^+^ in humans). Ly6C^lo^ monocyte “patrol” the luminal side of the endothelium of small blood vessels under homeostatic and inflammatory conditions (Auffray et al., 2007). Initial work suggested that the early phase after infarction is dominated by inflammatory (Ly6C^hi^) monocytes (Nahrendorf et al., 2007). They phagocytose toxic molecules and cell debris, have proteolytic activity and produce inflammatory cytokines like interleukin (IL)-1α, IL-6 and TNFα (Nahrendorf et al., 2007; Troidl et al., 2009; Woollard and Geissmann, 2010; Leuschner et al., 2012). Thus, by exercising inflammatory functions they are potentially harmful in this early phase and promote myocardial damage (Kaikita et al., 2004). In contrast to Ly6C^hi^ monocytes, non-classical Ly6C^lo^ monocytes were thought to play a role in the later stages after infarction (Nahrendorf et al., 2007). Through secretion of anti-inflammatory cytokines and growth factors, like VEGF and TGF-ß, they could promote angiogenesis, extracellular matrix synthesis and myocardial healing (Nahrendorf et al., 2007; Troidl et al., 2009). However, the clear categorization of monocyte subsets into distinct phases and functions after infarction has recently been challenged. Ly6C^lo^ monocytes are critically dependent on the transcription factor Nr4a1 for their development and survival (Hanna et al., 2011). Interestingly, absence of Ly6C^lo^ monocytes in Nr4a1-deficient animals does not abrogate the bi-phasic inflammatory response. These findings might be explained by the concept that Ly6C^lo^ cells derive from Ly6C^hi^ monocytes, which display high plasticity and develop into either proinflammatory or anti-inflammatory monocytes/macrophages (Hilgendorf et al., 2014; Ismahil et al., 2014). The transcription factor Nr4a1 thereby seems to serve as a master switch that controls the polarity of monocytes and could represent a potential target to modulate the local inflammatory response, which drives adverse cardiac remodeling. The findings also indicate that inflammatory Ly6C^hi^ monocytes are the predominant monocyte subset to be recruited to the infarct area not only in the first days of infarction but throughout the course of post-infarct remodeling (Nahrendorf et al., 2010; Hilgendorf et al., 2014; Ismahil et al., 2014). This is supported by data in humans demonstrating that peak levels of the corresponding subset of inflammatory (CD14^+^) monocytes correlate negatively with the recovery of left ventricular function after acute myocardial infarction (Tsujioka et al., 2009). In mice, inhibition of Ly6C^hi^ inflammatory monocyte recruitment into the infarcted myocardium by genetic ablation of C-C chemokine receptor type 2 (CCR2) reduced adverse cardiac remodeling (Kaikita et al., 2004).

Future studies are needed to clearly define the role and impact of monocyte subsets in the course of infarction. In any case, preclinical (Panizzi et al., 2010) and clinical data (Tsujioka et al., 2009; Aoki et al., 2011) indicate that monocyte/macrophage recruitment needs to be tightly controlled as dysregulated infiltration results in infarct expansion, left ventricular dilation, and heart failure. In detail, monocytosis disturbs resolution of inflammation in murine infarcts and consequently enhances left ventricular remodeling (Panizzi et al., 2010). This is associated with the extent of myocardial salvage and the recovery of left ventricular function after acute infarction (Tsujioka et al., 2009).

As a side note, neutrophils accumulate in larger numbers during the early phase of ischemic injury, but disappear within approximately 24 h whereas the monocyte/macrophage response is sustained (Jung et al., 2013; Nahrendorf and Swirski, 2013). In addition to the recruitment of blood derived monocytes, tissue-resident macrophages in the heart represent another source of innate immune cells, which likely contribute to a local inflammatory process in the ischemic myocardium (Epelman et al., 2014a).

## Origin of resident cardiac macrophages

**Figure 1:**
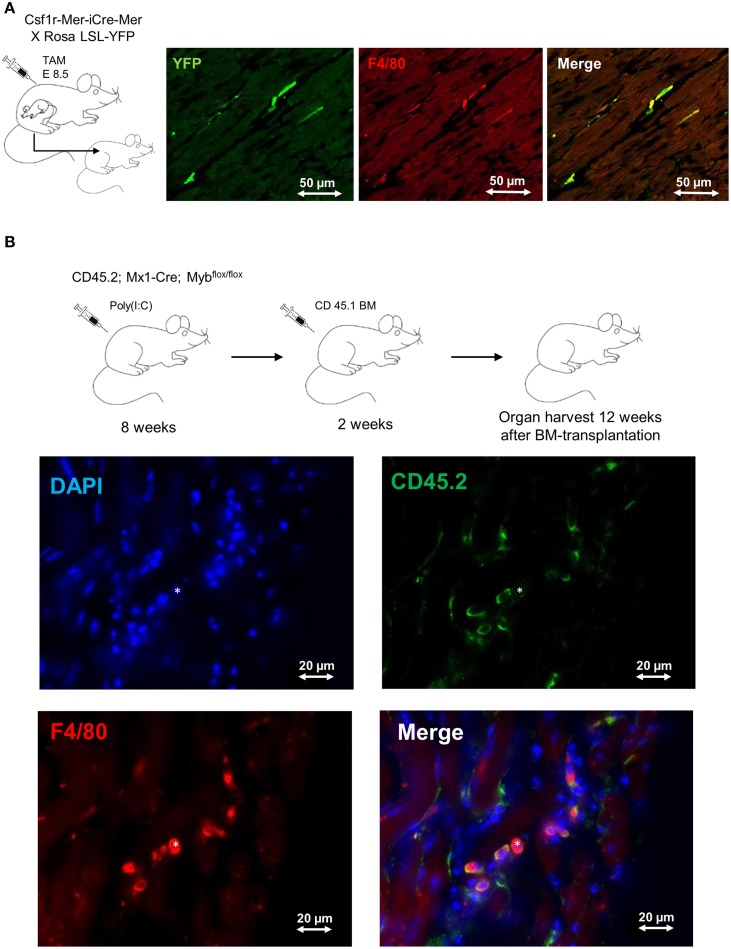
**Fate mapping analysis of cardiac macrophages**. **(A)** Frozen tissue sections of 1 year old mouse. FVB strain Csf1r^MeriCreMer^ females mated with BL6 ROSA ^LSL-YFP/LSL-YFP^ male were pulse labeled by injection of 75μg/g hydroxytamoxifen on embryonic day 8.5 (E8.5). This labeling regime allowed the specific labeling of Csf1r^+^ cells derived from the yolk sac, as definitive hematopoiesis is not present at that time point. 1 year old mice were stained for F4/80 (red) and YFP (green); double-positive cells represent yolk-sac derived macrophages. Bars 50 μm (Schulz et al., 2012; Epelman et al., 2014a). **(B)** Frozen tissue sections of 6 month old *Myb*-deficient BM chimera (CD45.1/CD45.2). BM ablation was obtained through deletion of *Myb* by injection of poly(I:C) into CD45.2; Mx1-Cre; Myb^flox/flox^ mice. Whole BM (10^7^ cells) from CD 45.1 mice was transplanted after BM-ablation as described earlier (Schulz et al., 2012). The heart was examined 12 weeks after BM transplantation and sections were stained with DAPI (blue), F4/80 (red) and CD45.2 (green); double-positive (F4/80 positive, CD45.2 positive) cells represent host-derived macrophages; (*) represents a donor-derived macrophage (F4/80 positive, CD45.2 negative). Bars 20 μm.

The formerly believed rational, that blood-derived monocytes are the only source of macrophages in the heart, has been challenged by recent work establishing the yolk sac as a common source of macrophages in adult tissues (Ginhoux et al., 2010; Schulz et al., 2012; Gomez Perdiguero et al., 2015). In line with these findings, also cardiac macrophages have their origin, at least to some extent, in the yolk sac (YS). Tamoxifen-induced pulse-labeling of Csf1r^+^ YS macrophages between embryonic days E8.5 and E9.5, when definitive hematopoiesis is not yet present, labeled a proportion of resident macrophages in the adult mouse heart (Epelman et al., 2014a; Molawi et al., 2014) (Figure 1A). These initial fate-mapping strategies were useful to assess the qualitative contribution of YS progenitors to adult tissue macrophages (Schulz et al., 2012; Epelman et al., 2014b). More recent work assessed the quantitative potential of YS progenitors for tissue resident macrophages *in vivo*, and established the YS as the key source of macrophages in adult tissues (Gomez Perdiguero et al., 2015). While YS-derived macrophages initially display a common signature (Schulz et al., 2012), their distinct phenotype and function is later shaped by local environmental cues specific for the respective tissue of residence (Gosselin et al., 2014; Lavin et al., 2014). Tissue macrophages perform specific duties, such as homeostatic functions and immune surveillance in other tissues (Davies et al., 2013). Nevertheless, their role in inflammatory processes in the heart needs to be determined.

It remains unknown to date, for how long YS-derived macrophages reside in adult tissues. In many organs, such as the brain and the liver, these cells are not replaced at steady state (Gomez Perdiguero et al., 2015) and can persist independently of HSCs (Schulz et al., 2012) (Figure 1B). However, a specific environment (e.g., intestine) or bacterial infections may result in replacement of tissue macrophages by bone marrow-derived monocytes (Bain et al., 2014; Bleriot et al., 2015). In the intestine, YS-derived macrophages are replaced by bone marrow monocytes within the first weeks of life due to substantial changes of the environment. Interestingly, the number of YS-derived resident macrophages in the mouse heart also seems to be impermanent and declines with age. As mice grow older the proliferation rate of cardiac macrophages decreases and is probably insufficient to maintain the resident macrophage pool (Molawi et al., 2014). Likewise, resident macrophages in the heart are lost as a consequence of myocardial infarction. They need to be substituted either through monocyte intermediates, which are recruited from the circulation, or by local proliferation and expansion of resident cells. Further experimental evidence is needed to define the quantitative contribution of circulation monocytes to the cardiac macrophage pool during aging and in the setting of myocardial injury (Heidt et al., 2014; Lavine et al., 2014; Molawi et al., 2014). Finally, it is still unknown to what extent monocyte-derived macrophages are distinct from resident macrophages with respect to their effector functions in tissues (Schulz and Massberg, 2014). Undoubtedly, the findings will have implications for our understanding of cardiac homeostasis and disease.

## Macrophage subsets in the heart

Recently, it has become clear that cardiac macrophages consist of several different subpopulations (Epelman et al., 2014a; Heidt et al., 2014; Lavine et al., 2014; Molawi et al., 2014). Elaborate multicolor flow cytometry studies allowed a comprehensive characterization of different macrophage subsets by cell surface markers including CD11b, F4/80, Cx3Cr1, major histocompatibility complex II (MHC II), CCR2 and Ly6C.

Accumulating evidence suggests that the composition of macrophage subsets in the myocardium undergoes dynamic changes in the course of life. This needs to be kept in mind when discussing cardiac macrophage populations in the heart, especially as the macrophage subsets have different regenerative and inflammatory potential. In newborn mice cardiac macrophages display a MHC II^lo^ phenotype and have predominantly originated from the yolk sac. This subset of macrophages seems to have distinct, probably beneficial, effects on the myocardium under stress. As mice age, the macrophage phenotype diversifies into a MHC II^lo^ and a MHC II^hi^ phenotype. Fate mapping analysis in adult mice using pulse labeling of macrophages indicated that the contribution of embryo-derived cardiac macrophages to the MHC II^lo^ subset was higher than to MHC II^hi^ macrophages. Between birth and 30 weeks of age resident macrophages become increasingly replaced by monocyte-derived macrophages from the bone marrow (Lavine et al., 2014; Molawi et al., 2014).

Another surface marker to describe macrophage subsets is CCR2, the receptor for chemokine monocyte chemoattractant protein-1 (MCP-1/CCL2), which facilitates monocyte chemotaxis. This marker helps to identity macrophages, which recently derived from circulating monocytes (Epelman et al., 2014a; Lavine et al., 2014). Using CCR2 and MHC II, cardiac monocyte, and macrophage subsets of the neonatal and adult heart could be defined. In a nutshell, at least two subsets exist in the neonatal heart at steady state, namely a CCR2^−^/MHC II^lo^ subset derived predominantly from the yolk sac and a CCR2^+^/MHC II^lo^ subset derived from definitive hematopoiesis. In contrast, in the adult heart (6 weeks of age), the monocyte/macrophage population could be divided into 4 subsets. The CCR2^−^ population had a diversified phenotype with MHC II^lo^ and MHC II^hi^ macrophage subsets and a mixed developmental origin. In contrast, the CCR2^+^ population derived exclusively from definitive hematopoiesis and could be subdivided into MHC II^hi^ and MHC II^lo^ populations (Lavine et al., 2014).

## Age-dependent response of macrophages to myocardial stress

Besides the unknown influence of the origin of macrophages on myocardial repair, it has become clear that macrophages exhibit a distinct potential to facilitate regeneration during different stages of life. Subjecting neonatal mice to myocardial infarction on postnatal day 1 (P1), the ischemic myocardium has been shown to completely regenerate by proliferation of preexisting cardiomyocytes. This regeneration capacity is already lost during the first week after birth. Induction of myocardial infarction 14 days postpartum (P14) showed left ventricular scarring and cardiomyocyte hypertrophy comparable to the pathophysiological changes seen in adult animals after myocardial infarction (Porrello et al., 2013). Macrophages differed in number, localization, and gene expression profile in mice subjected to myocardial infarction on P1 and P14 after birth. Even more strikingly, the regenerative capacity of the neonatal heart after myocardial infarction was lost when macrophages were depleted using clodronate liposomes. The depletion of macrophages in neonates was accompanied by a loss of neovascularization (Aurora et al., 2014). These results stress the importance of macrophages in the regeneration potential of neonatal mice.

**Figure 2:**
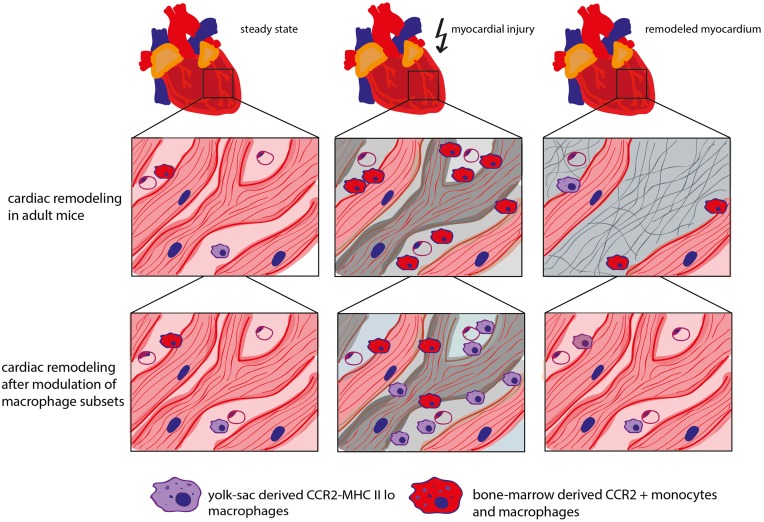
**Role of different macrophage subtypes in cardiac remodeling**. In steady state, the adult mouse heart contains various types of macrophage populations, which are of yolk sac (YS) or bone marrow (BM) origin dependent on the mouse age. CCR2^−^ macrophages are of mixed origin but predominately derive from YS/embryonic precursors. CCR2^+^ monocytes and macrophages are derived from the bone marrow. In adult mice, myocardial stress triggers the depletion of resident cardiac macrophages followed by the recruitment of “inflammatory” CCR2^+^ monocytes. Inhibition of CCR2 leads to a decreased recruitment of CCR2^+^ monocytes into the myocardium and protects resident cardiac macrophages which has a beneficial impact on coronary angiogenesis, inflammation and cardiac remodeling (Kaikita et al., 2004; Lavine et al., 2014).

In fact, neonatal mice react to myocardial stress (e.g., after *in vivo* cardiomyocyte cell ablation) with an expansion of the MHC II^lo^ macrophage subpopulation. It could be shown that these macrophages possess proangiogenic and reparative properties which, at least partially, contribute to the regenerative capacity of the neonatal heart. Fate mapping analysis revealed that this macrophage subset is of yolk sac origin. In contrast, adult mice respond to myocardial injury with the recruitment of inflammatory MHC II^hi^ CCR2^+^ macrophages from the blood circulation and loss of potentially beneficial CCR2^−^ (resident) macrophages. These mice developed pathological left ventricular remodeling including cardiomyocyte hypertrophy, fibrosis, and LV dilation resulting in a decreased LV function. Blocking the recruitment of CCR2^+^ monocytes into the injured myocardium preserved cardiac MHC II^lo^ macrophages in the adult heart and showed beneficial effects on coronary angiogenesis and attenuated inflammation (Lavine et al., 2014). This underscores a beneficial role of resident cardiac macrophages (Figure 2).

It is currently unknown whether the effects of the different macrophage populations on cardiac remodeling after *in vivo* cardiomyocyte cell ablation also apply to the myocardial infarction model. Intriguingly, myocardial infarction in adult mice is accompanied by the loss of resident cardiac macrophages. Consequently, blood-derived monocytes are recruited to the ischemic myocardium and repopulate the cardiac resident macrophage population (Heidt et al., 2014). Further studies are required to unravel the role of the different macrophage populations in infarct healing.

## Macrophages as therapeutic targets for myocardial repair

Monocytes and macrophages play a detrimental role in the pathophysiological processes triggered by infarction and therefore represent a potential therapeutic target to promote myocardial repair and functional regeneration. Yet we have only begun to understand the spatiotemporal relationships and functions of the different macrophage subsets in the course of infarct healing. It is likely that cell-type specific therapies as well as adequate timing of respective therapies will be able to achieve a more beneficial outcome in cardiovascular remodeling. In the following, we will highlight differential strategies targeting monocytes and macrophages, which have the potential to reduce the inflammatory burden in the post-infarct myocardium.

One approach that has been investigated for some time is blocking the recruitment of inflammatory monocytes. This can be achieved for example by interfering with chemokine gradients resulting in a reduction of circulating inflammatory monocytes (e.g., ablation of CCR2), or by inhibition of their recruitment into the ischemic myocardium (e.g., C-X-C chemokine receptor type 6 (CXCR6), macrophage migration inhibitory factor MIF). Reduction of inflammatory CCR2^+^ monocytes either using a knockout model of CCR2 or an antibody-mediated approach attenuated the inflammatory response after myocardial infarction and had beneficial effects on cardiac remodeling (Kaikita et al., 2004; Lavine et al., 2014). Likewise, disruption of the CXCL16-CXCR6 axis in an ischemia/reperfusion model, led to a decreased number of CD11b^+^ cells in the infarcted tissue resulting in improved cardiac function and attenuated cardiac remodeling through a diminished autophagic response (Zhao et al., 2013). Inhibition of MIF influences apoptotic pathways and other signaling cascades. Importantly, plasma MIF levels are associated with infarct size and extent of cardiac remodeling in humans. However, blockade of MIF is rather unspecific, and among other effects, also reduces the influx of neutrophils (Gao et al., 2011; Chan et al., 2013).

Another approach to modulate macrophage function is to modify their environment. As shown only recently, macrophage phenotypes and functions are shaped by the microenvironment of the organ of residence (Gosselin et al., 2014; Lavin et al., 2014). For example, transplantation of differentiated peritoneal macrophages into the lung environment induced reprogramming of the transcriptional landscape of these cells and their acquirement of new tissue specific functions. It is tempting to speculate that the same is true for the cardiac microenvironment.

Further, resident macrophages also interact with surrounding immune cells, including those of the adaptive immune system. Regulatory T cells have recently been shown to modulate monocyte/macrophage differentiation in the setting of myocardial infarction with a positive impact on wound healing and remodeling (Weirather et al., 2014). Whether enhancement of regulatory T-cells is beneficial for infarct repair is currently unknown. In addition, B lymphocytes interact with monocytes in the course of cardiac repair after myocardial infarction. In this setting, B cells facilitate mobilization and recruitment of Ly6C^hi^ monocytes into the ischemic myocardium by secretion of CCL7. Genetic as well as antibody-induced depletion of B cells reduced circulatory monocytes and inflammatory Ly6C^hi^ monocytes in the myocardium which improved cardiac function and attenuated myocardial injury (Zouggari et al., 2013). However, the precise mechanisms of the crosstalk between the adaptive immune system and monocytes/macrophages and its therapeutic potential in cardiac remodeling will require further experimental data.

Depletion of macrophages could represent another approach to modulate myocardial inflammation. However, this approach is likely to represent a double edged sword. Depletion of by injection of clodronate liposomes during the first week after myocardial injury led to reduced removal of necrotic cells, impaired neovascularization and increased scar formation. This resulted in a higher mortality of macrophage-depleted mice (van Amerongen et al., 2007; Ben-Mordechai et al., 2013; Frantz et al., 2013). Further, clodronate liposomes deplete most phagocytic cells, including dendritic cells (Leenen et al., 1998) and are therefore not specific. Still, phagocytic properties of mononuclear phagocytes may be harnessed to deliver drugs to their site of action, as reviewed elsewhere (Ben-Mordechai et al., 2015).

Finally, resident cardiac macrophages might be targeted through the alteration of intracellular signals, which for example induce proliferation or apoptosis. IL-4 has been shown to directly signal to tissue-resident macrophages and induce their proliferation above homeostatic levels (Jenkins et al., 2013). Targeting expression of the transcription factor MafB may inhibit macrophage apoptosis in tissues during inflammatory conditions (Hamada et al., 2014). Small-interfering RNA could thereby help achieve robust gene silencing. Delivery of respective particles in an encapsuled form revealed an efficient uptake by phagocytes (Aouadi et al., 2009). However, strategies targeting function of resident macrophages will have to account for their physiological role in tissue homeostasis (e.g., Nr4a1) in organs, including the heart (Schulz and Massberg, 2014).

In summary, modulation of macrophage functions represents an interesting therapeutic strategy. Its success, however, will depend on a deeper understanding of the heterogeneity of macrophage subsets and their developmental origin and molecular regulation. Further, the regulatory signals derived from the local microenvironment in the heart at steady state and in the course of infarction, which modulate the phenotype and functional specification of macrophages, require further investigation.

## Concluding remarks

Despite their abundance in the myocardium, the function of the different cardiac macrophage populations at steady state and in response to stress is largely unknown. There is no doubt that future studies will reveal the precise contribution of monocyte/macrophage populations to myocardial remodeling in pathological conditions thereby integrating findings on their developmental origin and regulation. Of note, recent data in mouse models of infarction suggest that “time” may matter in various ways. First, monocyte/macrophage populations may have a distinct role at different stages of myocardial remodeling and thus, targeting these immune cells at specific time points could further improve wound healing and functional outcome. Second, it may also play a role at which age experimental models (e.g., myocardial infarction) are performed, as macrophage populations in the heart are –at least in mice– replaced over time (Molawi et al., 2014) and also the recovery potential abates with age (Lavine et al., 2014). These recent studies on the heterogeneity and kinetics of monocytes and macrophage subsets in the mammalian heart have paved the way for new therapeutical strategies to target infarct repair after myocardial infarction. Despite the recent advancement on our understanding of the pathomechanisms, specifically the kinetics and regulation of inflammatory processes and wound healing in the diseased heart, early reperfusion therapy for patients who have myocardial infarction remains the essential basis to preserve myocardial function and reduce mortality.

### Conflict of interest statement

The authors declare that the research was conducted in the absence of any commercial or financial relationships that could be construed as a potential conflict of interest.
